# Finite Element Stress Analysis of Keyhole Plate System in Sagittal Split Ramus Osteotomy

**DOI:** 10.1038/s41598-018-27186-7

**Published:** 2018-06-12

**Authors:** Ju-Won Kim, Kang-Nam Park, Chang-Hyeon Lee, Yong-Su Kim, Young-Hee Kim, Byoung-Eun Yang

**Affiliations:** 1 Department of Oral and Maxillofacial Surgery, Hallym University College of Medicine, Anyang, South Korea; 2 Department of Image Science in Dentistry, Hallym University College of Medicine, Anyang, South Korea; 3 Graduate School of Clinical Dentistry, Hallym University, Chuncheon, South Korea; 4 Institute of Clinical Dentistry, Hallym University, Chuncheon, South Korea

## Abstract

A new miniplate applied differently from conventional application method for bone fixation has been developed. The novel approach is the insertion of the screw into the bone before miniplate installation. This study aimed to assess the stress distribution of a newly designed Yang’s Keyhole (YK)- plate for segmental-bone fixation during sagittal split ramus osteotomy (SSO). Moreover, the effectiveness of the YK-plate system based on the clinical results was determined. The YK-plate system has a widened hole in the anterior region to permit a screw-head to be screwed through the system. The stress distribution using the finite-element analysis method was compared between in the case of the YK-plate system and the case of existing mini-plate fixation technique. Moreover, the clinical results of patients were evaluated during the follow-up examination periods. No critical complications in any of the six patients were reported during the four-month follow-up period. The result of the stress distribution using finite-element analysis showed a similar trend in all four fixation methods. The YK-plate system can be applied to fixation during SSO and allow for mechanically stable and convenient application.

## Introduction

Orthodontic treatment for a patient with craniofacial deformities and facial asymmetry has clinical limitations for achieving reliable outcomes. Sometimes, patients need to undergo orthognathic surgery combined with orthodontic treatment. Since the first introduction of a sagittal split ramus osteotomy (SSO) in 1957 for a maxillofacial deformity, substantial progress has been made in the surgical procedure^1,2^. Generally, bone segment fixation methods after SSO include the use of a wire, bicortical screw, and mini-plate with a mini-screw^3–8^. Currently, in many cases, a mini-plate with a mini-screw is used to fix the bone segment after SSO, and some newly designed plates have been introduced for postoperative stability and the convenience of surgery^3–7^. Miniplate was first introduced by Michelet in 1973 and further modified by Champy in 1975^9^. Since then, various types of mini-plates have been developed and used for craniofacial surgery. The use of a mini-plate in craniofacial bone surgery was routine, and the method of application was similar even though the shape or form of the plate was different. The mini-plate to be reported in this study is similar in shape to the existing ones. However, this mini-plate has been designed to be applied in a different order from the conventional sequence, and clinical efficacy has been reported in the case of 22 patients with mandibular fracture reduction^10^. One of the multiple holes on miniplate is in the form of the keyhole, and we named it Yang’s Keyhole(YK)-plate. The insertion method is as follows. First, the screw is inserted into the bone hole approximately 1/2 of its length into the bone hole. The keyhole part of the YK- plate is then adjusted by sliding it after inserting it into the head of the screw first placed. Next, the standard head size screws are inserted into the remaining holes: the screw first inserted is fully inserted in depth, and the oversized head screw is inserted into the remaining large part of the keyhole. We reported the effectiveness of YK-plate in the treatment of mandible fractures^10^. An examination of surgical outcomes on a mandible replica showed that the YK-plate system, the benefits of which are obtained by changing the order of the system’s application as the shape of hole of the sliding-plate is changed. Besides, the convenience felt by a surgeon during surgery can be significantly increased for surgeons inexperienced in maxillofacial bone surgery^10^. Therefore, we applied this plate to orthognathic surgery and similarly to the fracture reduction surgery; it was expected that the convenience of application and the shortening of time would be achieved in orthognathic surgery. However, since the YK-plate had a different shape from the conventional plate, it was necessary to evaluate which part of the YK-plate was stressed when the load was applied. It is controversial to identify the best design and configuration of titanium plates to maximized fixation stability^11^. Finite Element Analysis (FEA) is one of the numerical techniques that simulate the mechanical aspect of a structure under load^12^. FEA and Von Mises analyzes are widely known to be useful for generating the virtual models of biomedical instruments and for evaluating stress distributions in critical areas^13^. This method is used to study mechanical aspects of biomaterials and human tissues. FEA has been widely used to predict the effect of stress on biomaterials and its surrounding structure for the last 30 years^14^. In this study, to confirm the mechanical stability of the YK-plate, we analyzed and compared the distribution of stresses according to the clinical loading conditions by FEA for conventional fixation with a mini-plate, fixation with a sliding-plate or the YK-plate after creating the SSO model. The authors also reported that the YK-plate was applied to six patients who underwent SSO.

## Results

### Finite element analysis

**Figure 1:**
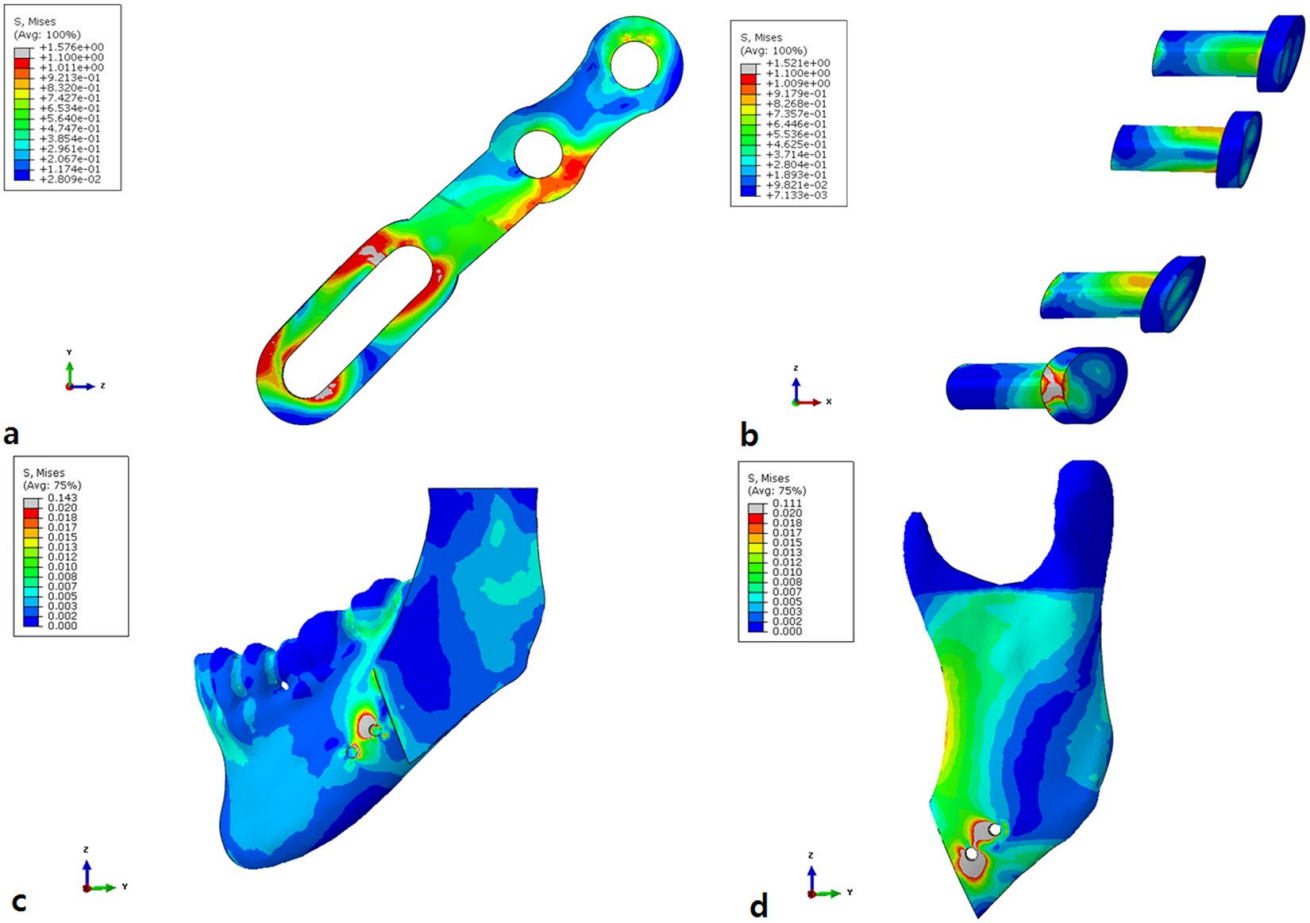
Finite-element model demonstrating stresses in the sliding-plate model. (**a**) Sliding-plate, (**b**) Four screws, (**c**) Distal segment (buccal view) and (**d**) Proximal segment (buccal view).

**Figure 2:**
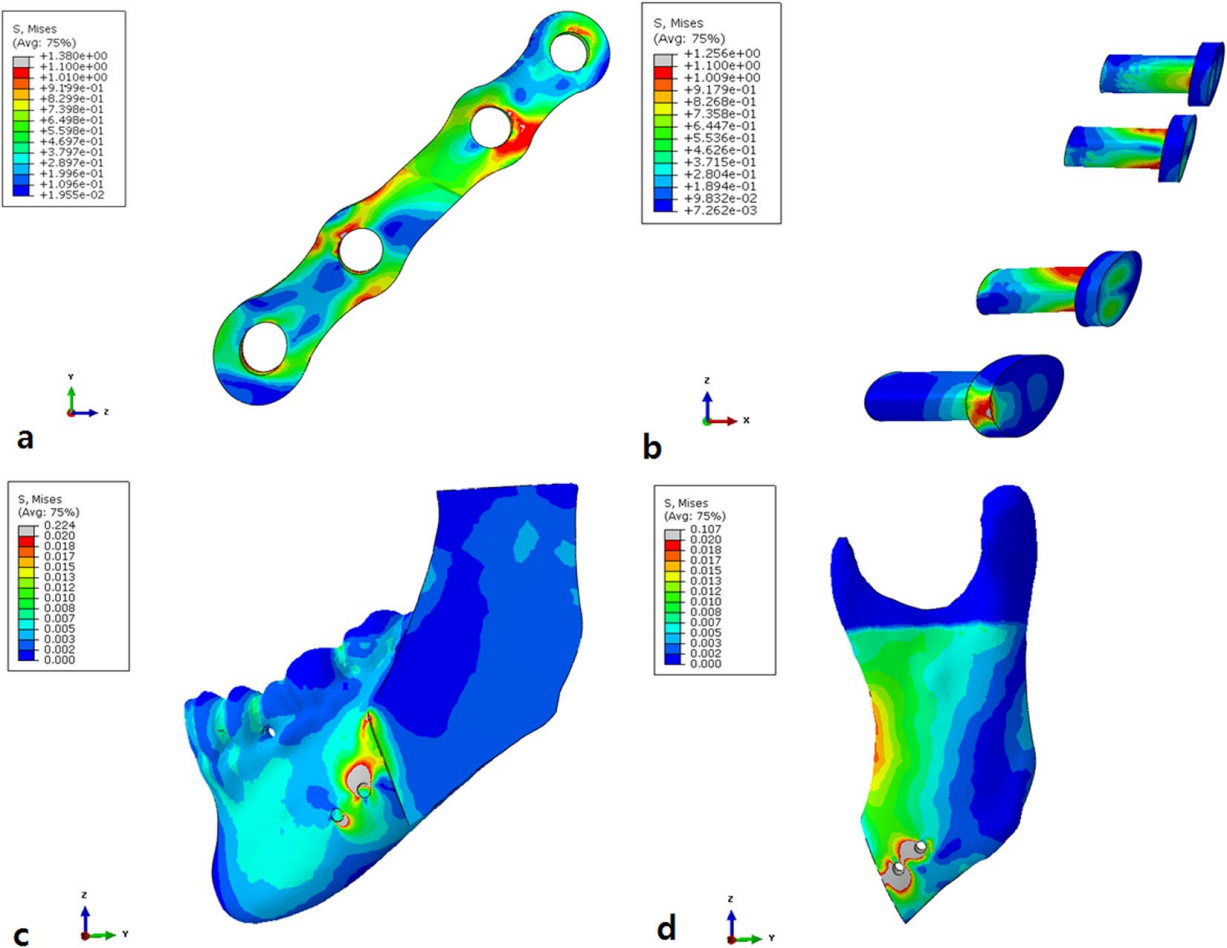
Finite-element model demonstrating stresses in the 4-hole bridge plate model. (**a**) Four-hole bridge plate, (**b**) Four Screws, (**c**) Distal segment (buccal view) and (**d**) Proximal segment (buccal view).

**Figure 3:**
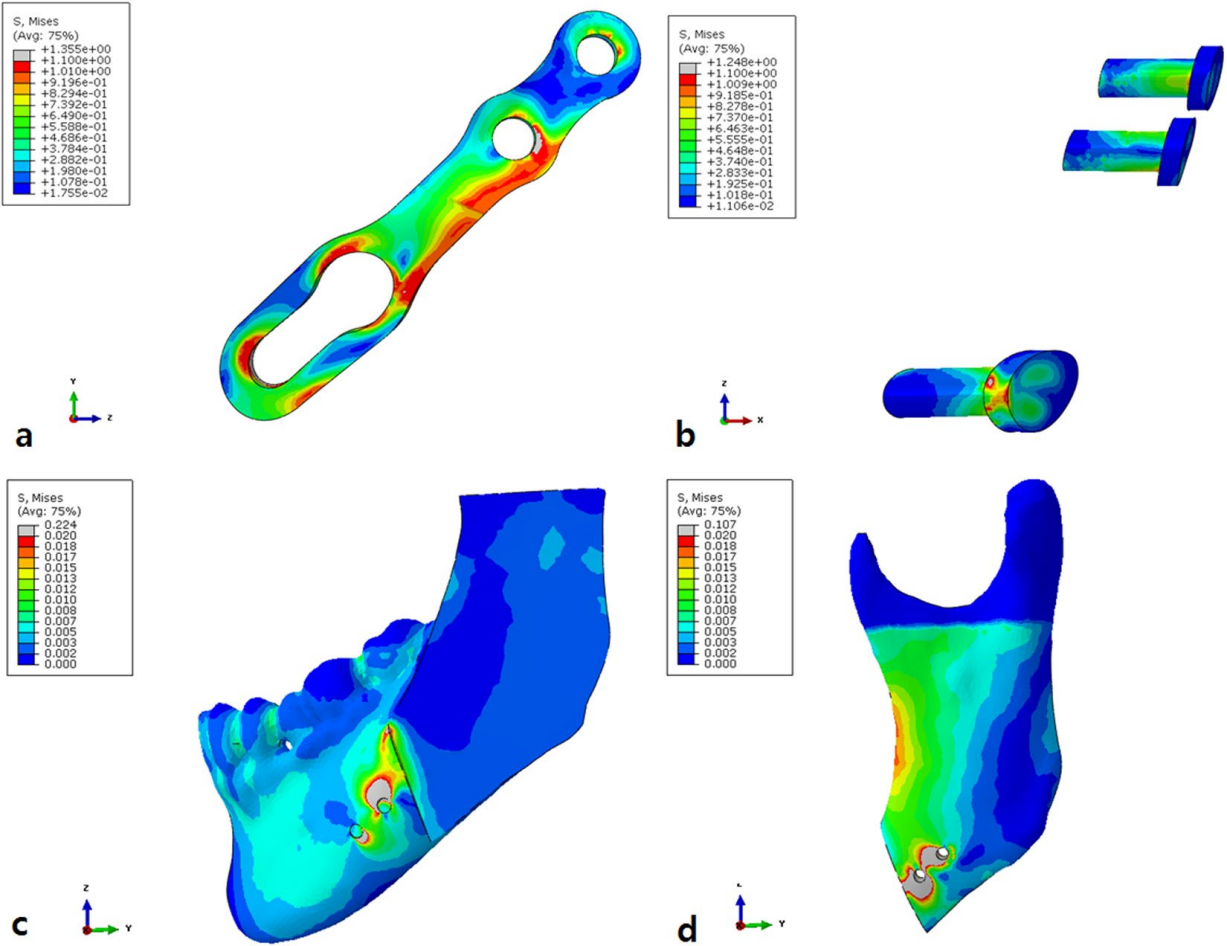
Finite-element model demonstrating stresses in the YK-plate model. (**a**) YK-plate, (**b**) Three screws, (**c**) Distal segment (buccal view) and (**d**) Proximal segment (buccal view).

**Figure 4:**
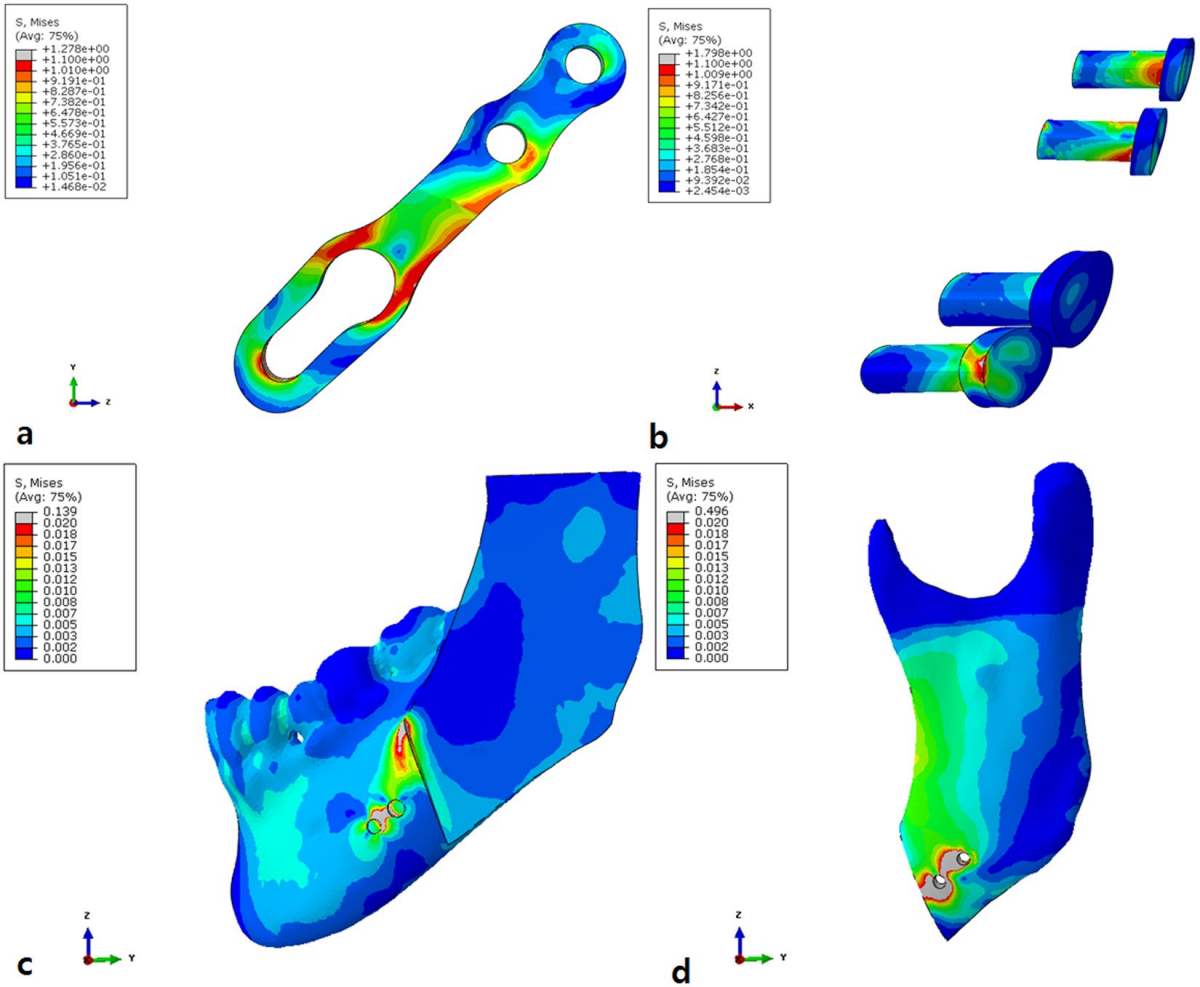
Finite-element model demonstrating stresses in the YK-plate with the oversized head screw model. (**a**) YK-plate, (**b**) Three screws and one oversized head screw, (**c**) Distal segment (buccal view) and (d) Proximal segment (buccal view).

After a uniform force was applied to all models, the distribution of stresses in the mandible, the mini-plate, and the screw was as follows. The maximum principal and general effective stresses were evaluated for each model. And for the property analysis, the color grading was used to indicate the stress value of 13 steps. In case of the sliding-plate, according to the FEA, the maximum principal stress was observed around the screw near the osteotomy line, and the minimum stress distribution was found around the most anterior screw. A high-stress concentration was seen in the long hole area of the anterior part and the second mini hole, and the highest stress was observed at the anterior portion of the long hole (Fig. 1). In the case of a four-hole bridge plate, according to the FEA, the maximum principal stress was observed around the screw near the osteotomy line, and the minimum stress distribution was found around the anterior screw. The plate showed a relatively uniform stress distribution with the highest stress in the screw hole around the osteotomy line (Fig. 2). In the case of the YK-plate with three screws, according to the FEA, maximum principal stress was observed around the osteotomy line on the proximal segment, and the minimum stress distribution was found around the anterior screw. The YK-plate showed high-stress concentration under the bridge area and the highest stress in the most anterior part of the YK (Fig. 3). The effective general stress of the YK-plate with three screws and oversized-head screw obtained using FEA was observed near the proximal segment of the osteotomy, and the minimum stress distribution was located on the screw in the anterior region. The YK-plate showed a high-stress concentration at around the bridge in the posterior region, and the highest stress concentration was located in the anterior region of the keyhole part. The YK-plate showed a stress distribution pattern similar to that with the placement of an additional oversized-head screw, but the degree of stress distribution on the mandible and YK-plate decreased (Fig. 4).

### Clinical Evaluation

**Figure 5:**
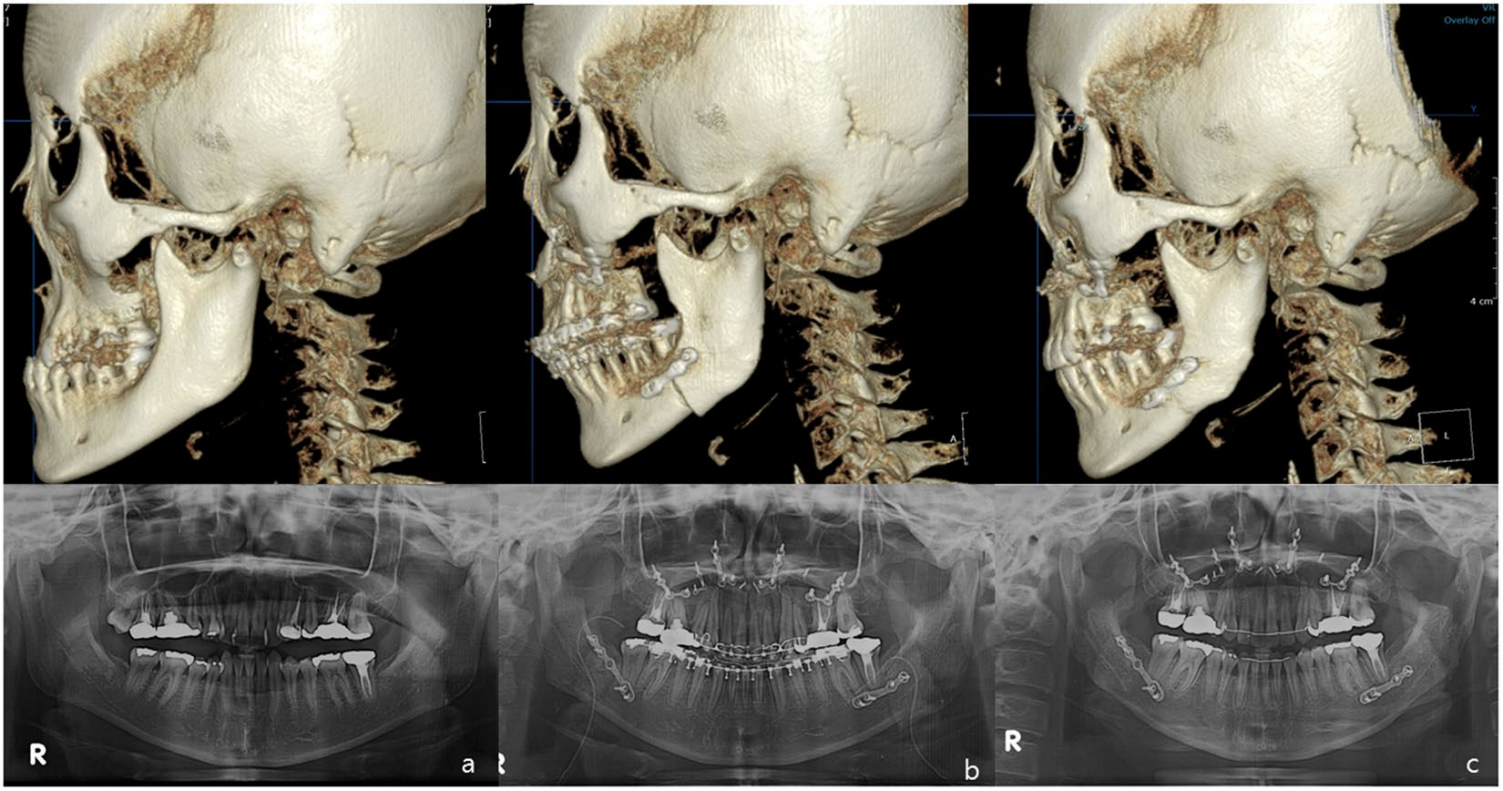
Three-dimensional ConeBeam CT and Panoramic radiographs demonstrating the use of a YK- plate after SSO. (**a**) Preoperative view, a 23-year-old patient with mandibular prognathism (**b**) 2 days after the operation, Bimaxillary operation was done. YK-plates were used on mandible (**c**) four months after the surgery, the bone remodeling is in progress, and the YK-plates are stable.

Complications, such as occlusal instability, inflammation, nerve abnormality, malunion, or plate breakage, were not observed in the six patients (Table 1). All patients were stable postoperatively for four months without relapse (Fig. 5). Clinical evaluation showed that the use of the YK-plate in orthognathic surgery did not cause unexpected complications or problems.

**Table 1:** Clinical outcome with the checklist during follow up.

**The total number of patients**	**6**
Relapse, malocclusion	None
Temporomandibular joint disorder (pain, noise and mouth opening)	1/6 (temporary three weeks)
Nerve disorder (inferior alveolar nerve, lingual nerve and facial nerve)	None
Postoperative infection	None
Malunion, non-union	None
Screw loosening or plate fracture (clinical and radiographic evaluation)	None
Operative availability (subjective)	good (subjective)

## Discussion

**Figure 6:**
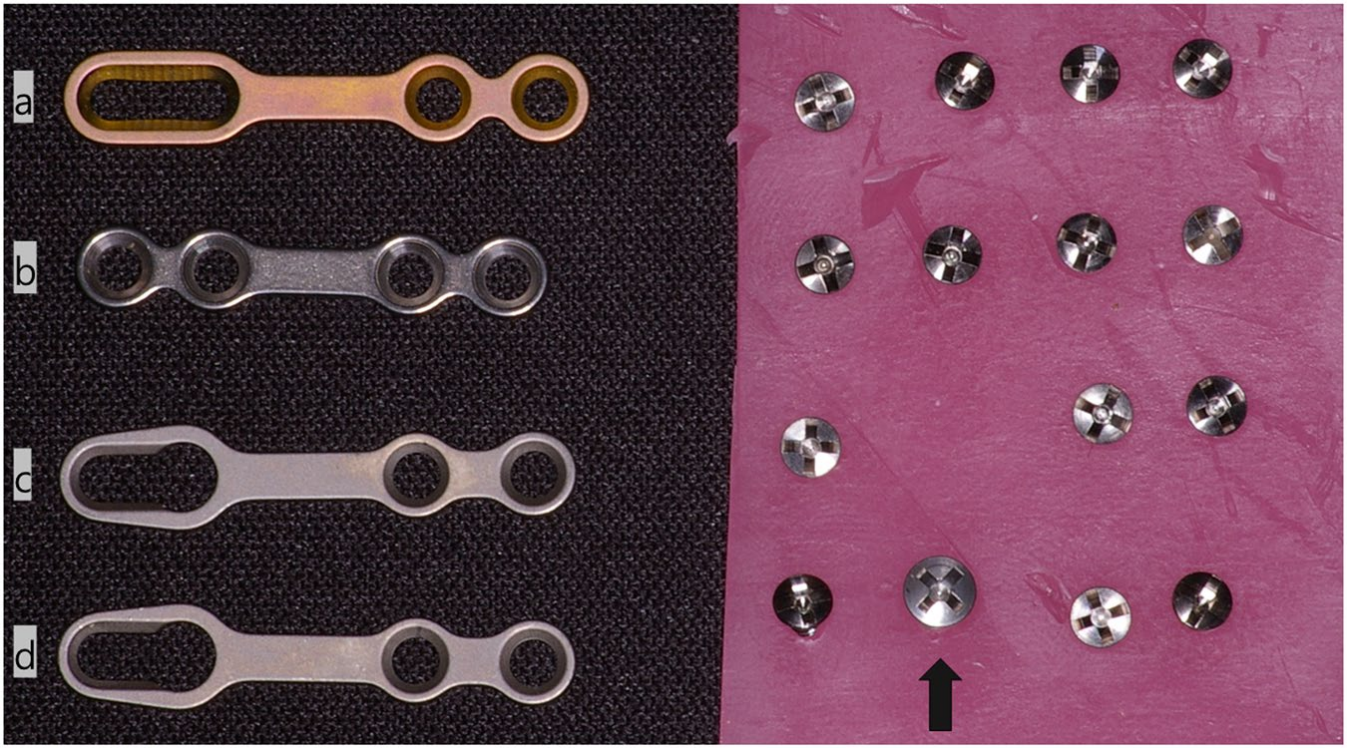
Four types of fixation after SSO. (**a**) Sliding-plate with four screws, (**b**) Four-hole bridge plate with four screws (**c**) YK-plate with three screws and (**d**) YK-plate with three screws and one oversized head screw (black arrow).

The use of the YK-plate in a mandible fracture can quickly reduce the fractured bone segment and fix it to the correct position according to the surgeon’s intention by placing an anchor screw in the wide hole at the anterior part^10^. In orthognathic surgery, the YK-plate can be used to stabilize the condylar position with a small modification of the anterior hole, such as a sliding-plate, and it is possible to adjust the fixation rigidity according to the placement of the oversized head screw^15,16^. Generally, the most common problem after SSO is condyle sagging. Condyle sagging brings about open bite and relapse after surgery^17^. Sliding-plates allow the movement of the proximal and distal segments and help stabilize the condyle postoperatively^18^. The YK-plate can also maintain the condyle position through a wide anterior hole. Additionally, if postoperative radiological and clinical examinations show that the occlusion is stabilized, and the condyle is in a stable place, it is possible to obtain long-term stability by the self-tapping oversized head screw placement through stab incision under local anesthesia. The purpose of this study is to investigate the difference between the newly designed YK-plate and the existing titanium plates using the FEA method. Since YK-plates are different in shape from conventional titanium plates, we had to investigate whether there were any problems with strength or durability. FEA is a method of analyzing the mechanical properties of a complex structure through a simple linear model and analyzing the interactions between structures. FEA allows us to evaluate the complex mechanical aspects that are affected by the mechanical loading of the mandible. FEA gave a good qualitative contribution in this sense being many variables in play^13^. The knowledge of the distribution of stress and strain, in the mandible and bone plates, is fundamental for the evaluation of adequate stability of the plates itself. In this study model, the stress distribution around the plate was estimated by assuming the masticatory force after SSO. Our FEA study was performed on a mandibular setback model. Mandibular setback surgery is performed more often than mandibular advance surgery in Asians. For it is reported that the prevalence of mandibular prognathism is highest in Asian populations (approximately 15%) and lowest in Caucasian populations (1%)^19,20^. The distribution of stress is similar in all the four cases. In the mandible, the highest stress was observed around the screw where the osteotomy was performed, and the plate showed the highest stress near the osteotomy line. The extent of the plate exceeding the stress limit was similar in all the four cases and mainly occurred in the anterior hole of the plate. The highest shear stress was observed near the screw in all the cases. One of the problems that can be expected from the YK-plate is the structural weakening caused by the formation of oversized hole sites. The oversized hole can lead to a breakage of the plate at the weakened site. FEA showed that the YK-plate had the highest stress distribution at the bridge connection instead of in the oversized hole area, and this area was not the area with the stress limit under continuous stress. Another likely problem is the displacement of the screw at the oversized hole site. In an oversized hole, the screw head is seated on the plate and displacement is not caused because it is movable following the internal shape of the hole (Fig. 6c and d). In this way, the YK-plate shows stability similar to that of the conventional mini-plate and can have the advantage of a conventional mini-plate. Besides, it can exhibit a similar effect as the sliding-plate and can be firmly fixed depending on whether the oversized head screw is placed. Analysis of the stress distribution of the YK-plate shows that similar results and effects can be obtained after the SSO using a conventional plate, and it can be used as an alternative fixation method after SSO. The sliding-plate has demonstrated advantages in various articles^15,16^. The YK-plate is thought to be easier to apply in clinical practice while taking advantage of the sliding-plate^15^. None of the six patients who participated in this 4-month retrospective clinical study had significant complications. The transient altered sensation was caused by the orthognathic surgery process itself instead of by the plate. The stages of the remodeling cycle of bone have different time lengths. Resorption probably continues for about two weeks, and the reversal phase may last up to 4 or 5 weeks, while formation can continue for four months until a new bone structural unit is completely created^21^. Therefore, if four months have passed since the bone surgery, the role of the titanium plate used to fix the bone can be decided to be over. Titanium plates can be evaluated as stable if no specific complications have occurred by this time. Although the number of patients we retrospectively studied was small and more patients needed for the evaluation of long-term stability, mechanical stability was not significantly different from that of other plates with FEA. Clinically, when they were used in facial bone fractured patients, good results were obtained^10^. Although the observation was made on a few cases and without comparative evaluation with other plates, the application time of YK-plates is shorter in orthognathic surgery than in conventional plates, as is the case for fractured patients. The YK-plate system with these advantages will be an alternative to other mini-plates for maxillofacial bone surgery, such as facial bone fracture and orthognathic surgery. In conclusion, the YK-plate system has the following advantages in fixation after SSO. First, stable condyle seating is possible by allowing the physiological movement of the proximal and distal segments after surgery like in the case of using sliding-plate. Second, the clinical application time is reduced compared to other titanium plates^10^. Lastly, additional fixation is possible via stab incision, if necessary, after appropriate clinical and radiological evaluation during or after surgery. In this study with FEA, YK-plates showed good mechanical stability in SSO as well as in conventional plates. The force acting on the mandible is not simple, and it is difficult to find out the various dynamic movements and forces of the living body using only the FEA used in our research. However, our study is a relative comparison between four fixation methods under the same conditions. Additional studies are needed which take into account various situation in the human body. The authors also suggest that it is necessary to study the indications of YK-plates via various approaches through modification, such as with an L shape or T shape or through changing the position or hole length of keyhole part. And the authors hope that the YK-plate will be widely used in craniofacial surgery.

## Methods

### YK-plate and Surgical Technique

The YK-plate (Osteonic Co, Seoul, South Korea) is a modification of the sliding-plate. There is a hole in the plate through which the screw head can pass. The sliding-plate consists of two round holes and one oval hole, providing space for adjusting the position of the proximal and distal bone segments^15,16^. The YK-plate also allows for movement of the proximal and distal segments through the anterior hole. The YK-plate consists of grade 2 pure titanium. It has a 3.5-mm wide hole on the anterior part, allowing the screw head to pass through, and two 2.2-mm holes on the posterior portion. Through this wide anterior hole, the screw can be fixed to the bone segment, and the plate can be applied. Additional stability is obtained by placing an extra oversized head screw (black arrow in Fig. 6) in this hole^10^. The YK-plate in a mandible fracture can quickly reduce the fractured bone segment by placing the anchor screw in the wide hole at the anterior part^10^. Orthognathic surgery, on the other hand, allows for the movement of the proximal and distal segments through the anterior hole, and an oversized screw can be placed in the anterior hole to obtain firm fixation.

### Finite element analysis

The DICOM format file obtained from Computed Tomography(CT) images of adult male mandibles was converted to an igs file and was used for analysis in Solid Works, a 3-dimensional computer-aided design software. We generated 3D elements to be applied to the analysis based on the 3D model created by Solidworks (Hypermesh v11.0). FEA was performed using commercial FEA software with the generated 3D finite element model (Abaqus/Explicit v6.10). Unilateral mandible models were used for simulation of SSO. The models were divided into proximal and distal segments after SSO, and 5 mm of the setback was performed. Then, the interference between the proximal and distal segments was eliminated. The mini-plate and screw were generated using the Solidworks program based on the manufacturer’s design. The mandibular shape is complex, with the C3D4 (4 node tetrahedron) element type used in the mandible. For the titanium plate and screws, the C3D8 (8 node brick) element type is used to improve the accuracy of the analysis. The elements and nodes are shown in Table 2.

**Table 2:** Finite element model elements and nodes.

Model	Elements	Nodes
Sliding plate with 4 screws	549,982	134,371
Four-hole bridge plate with 4 screws	535,831	133,536
YK-plate with 3 screws	377,229	99,831
YK-plate with 4 (3 + 1*) screws	578,010	142,240

*Oversized head screw.

The finite element model assumes uniform and linear elasticity and has the mechanical properties of Young’s modulus and the Poisson ratio. For the mandible, according to previous studies, Young’s modulus was 624.42 MPa, and the Poisson ratio was 0.28^22^. Since the mini-plate and screw consisted of pure titanium, Young’s modulus and Poisson ratio were applied as 116,000 MPa and 0.34, respectively^22,23^. In some studies, Young’s modulus values are set lower^24^ or higher^18,25^ than Young’s modulus values adopted in our study. However, the material properties of bone vary considerably from one individual to another and are impossible to determine *in vivo*. It is known that bone is an inhomogeneous, anisotropic material, and the study reports a wide range of values for material parameters^26^. In general, Young’s modulus of compact (cortical) bone can vary between 10 and 20 GPa, depending on, for instance, the bone architecture and the type of loading. In the case of cancellous bones, Young’s modulus is reported to be lower. The values of Young’s modulus of cancellous bones were found to range from 0.76 to 20 GPa, depending on trabecular density and orientation^27,28^. However, if the mandible is assumed to be homogenous for FEA, it has to be decided whether to Young’s modulus value of cortical bone or that of cancellous bone. Instead, our study adopted Young’s modulus value in polyurethane mandible model, which is similar to Young’s modulus value of cancellous bone^25,26,29^, which are presented in many other studies. There is no cortical bone connection immediately after SSO in a patient, and only the cancellous bone of the proximal segment and the distal segment is connected using a titanium plate. Therefore, when FEM analysis is done, it seems appropriate to use Young’s modulus value of cancellous bone. Under the premise that the proximal segment and condyle were fixed, a force was applied to the center of the second mandible molar to create a displacement of 3 mm. Through this finite element model, we compared between the four fixation methods (Fig. 6).

### Patients

Between January 2015 and December 2015, segmental-bone fixation was performed using the YK-plate after SSO in six patients (four females and two males; average age, 30.5 years; range, 23 to 49 years) at the department of oral and maxillofacial surgery of university hospital, and the cases were reviewed.

This study was conducted in accordance with the World Medical Association Declaration of Helsinki on medical research ethics. The approval of this study was granted by the Institutional Review Board of Hallym University Sacred Heart Hospital (IRB No. 2015-I058) and informed consent were obtained for this study. All patient data were anonymized and de-identified prior to analysis. Follow-up clinical evaluation was performed at weeks 1, 2, 4, 8, and 16 after surgery. Follow-up panoramic radiography and cone-beam computed tomography scans were performed for radiological evaluation two days and four months after SSO. Patients’ medical conditions, such as pain, occlusion, temporomandibular disorder, opening restriction, mandibular movement, nerve abnormality, inflammation, and oral hygiene, were examined and recorded by a single investigator during follow-up. The surgeons subjectively appraised the operative availability of the use of the YK-plates.

### Data availability

The datasets generated during and/or analyzed during the current study are available from the corresponding author on reasonable request.

### Ethical approval and informed consent

Retrospective clinical study was approved by Hallym University Sacred Heart Hospital’s institutional review board (IRB No. 2015-I058). All clinical study was performed in accordance with the relevant guidelines and regulations. Retrospective unnamed data collected for the evaluation purpose.
